# Uptake of plant-derived specific alkaloids allows males of a butterfly to copulate

**DOI:** 10.1038/s41598-018-23917-y

**Published:** 2018-04-03

**Authors:** Keiichi Honda, Junya Matsumoto, Ken Sasaki, Yoshiaki Tsuruta, Yasuyuki Honda

**Affiliations:** 1 Department of Biofunctional Science and Technology, Graduate School of Biosphere Science, Hiroshima University, Higashihiroshima, 739-8528 Japan; 2 Graduate School of Agriculture, Tamagawa University, Machida, 194-8610 Japan; 3 Present Address: Saijo Ecology Institute, 1387-38 Iida, Hachihonmatsu, Higashihiroshima, 739-0141 Japan

## Abstract

Certain butterflies utilize plant-acquired alkaloids for their own chemical defense and/or for producing male sex pheromone; a trait known as pharmacophagy. Males of the danaine butterfly, *Parantica sita*, have been reported to ingest pyrrolizidine alkaloids (PAs) as adults to produce two PA-derived sex pheromone components, viz. danaidone (major) and 7*R*-hydroxydanaidal. We found, however, that not all PAs that can be precursors for the pheromone serve for mating success of males. Here we show that although the sex pheromone is regarded as a requisite for successful mating, uptake of specific PA(s) (lycopsamine-type PAs) is also imperative for the males to achieve copulation. The increase in the levels of two biogenic amines, octopamine and/or serotonin, in the brain and thoracic ganglia of males fed with specific PA(s) suggested that these alkaloids most likely enhance male mating activity. The results can present new evidence for the evolutionary provenance of pharmacophagous acquisition of PAs in PA-adapted insects.

## Introduction

Animals not only require nutrients for growth and development but occasionally or routinely ingest particular phytochemicals for the purpose of self-medication, chemical defense against predatory enemies, or pheromone biosynthesis. The behavioural/physiological trait whereby animals ingest non-nutritive substances for particular purposes is known as pharmacophagy^1^, which is widespread in the animal kingdom. In insects, exploitation of pharmacophagously ingested chemicals has been discussed in light of self-medication^2^, for example, in honey bees^3^, monarch butterflies^4^, and a moth species^5^, of chemical defense in many lepidopteran taxa^6^, Orthoptera^7,8^, and Coleoptera^9,10^, and of the production of sex pheromones in Lepidoptera^11,12^, Hymenoptera^13^, Diptera^14^, and Neuroptera^15^. Among the most intensively and extensively studied plant chemicals that are involved in insect-plant interactions are pyrrolizidine alkaloids (PAs), which are well-known hepatotoxic plant secondary (specialized) metabolites that play diverse roles in terrestrial ecosystems and are closely associated particularly with arctiid moths and butterflies in the subfamilies Danainae and Ithomiinae (Nymphalidae)^11,16,17^. In these PA-adapted lepidopterans, PAs, acquired by larvae or adults from host or non-host plants, are utilized as precursors for the biosynthesis of male sex pheromones^18–21^ and for chemical defense against predators, parasitoids, or pathogens^22,23^.

Adults, particularly males, of most danaine butterflies have been observed in the field congregating on dead or decaying parts or flowers of plants that contain PAs (mainly Boraginaceae, Asteraceae and Fabaceae families)^24,25^, from which they imbibe the alkaloids. In most species, attraction to PA sources is strongly biased toward males^24^, probably because males would require PAs as precursors for the production of sex pheromones^18,21^. Males of most danaine butterflies have two types of androconial organs, viz. a pair of eversible abdominal hairbrushes (hairpencils) and a pair of alar glands (sex brands)^26,27^. The hairpencils are extruded and splayed nearby the female at a species-specifically programmed time point during precopularoty pursuit or hovering. The hairpencil chemicals are disseminated at the time of hairpencilling and simultaneously perceived by females^21,28^. Danaidone (DO) and hydroxydanaidal (HD) are PA-derived androconial components encountered in many danaines^29^. Of these, DO has been shown to play a significant role, as the male sex pheromone, in the courtship of *Danaus gilippus*^30,31^ and *Idea leuconoe*^20^.

Males of *Parantica sita* (Nymphalidae; Danainae), endowed with a black, relatively large patch-like sex brand situated near the anal angle of the hindwing, actively seek and preferentially ingest lycopsamine-type PAs from some boraginaceous and asteraceous plants (Supplementary Figure S1). We have previously shown that the males possess DO as a major component along with a much smaller amount of 7*R*-HD (7*R*-enantiomer of HD) in both the sex brand and hairpencil, and further demonstrated that either or both of these compounds act as the sex pheromone^21^. *P. sita* males often rub their half-expanded hairpencils against the alar patches prior to the commencement of courtship behaviour^11,32,33^. With regard to the formation of DO in the two androconial organs, we have found that DO is produced exclusively in the sex brand and subsequently physically transferred to the hairpencil through a contact behaviour between the two organs. This behaviour (termed perfuming) is a prerequisite for the occurrence of DO in the hairpencil. DO is biosynthesized from various PA precursors, while 7*R*-HD is derived only from PAs with the 7*R*-configuration^21,34^; in fact, the production of DO and 7*R*-HD heavily depends, in both quality and quantity, on the chemical structures of ingested PAs.

Based on these findings, we first tested which structural types of PAs most favor male mating success in *P. sita* in terms of the production of pheromone components. This study further examined how PA uptake by males affect their courtship behaviour, focusing on their activity in the sequence of precopulatory aerial interactions with females, and determined the levels of biogenic amines (known to function as neurotransmitters, neuromodulators, or neurohormones^35–37^) present in the brain and thoracic ganglia of males to deduce possible relationships between PA uptake and male mating success.

## Results

We conducted three behavioural experiments to test the effect of PAs on the mating success and courtship behaviour of males.

**Figure 1:**
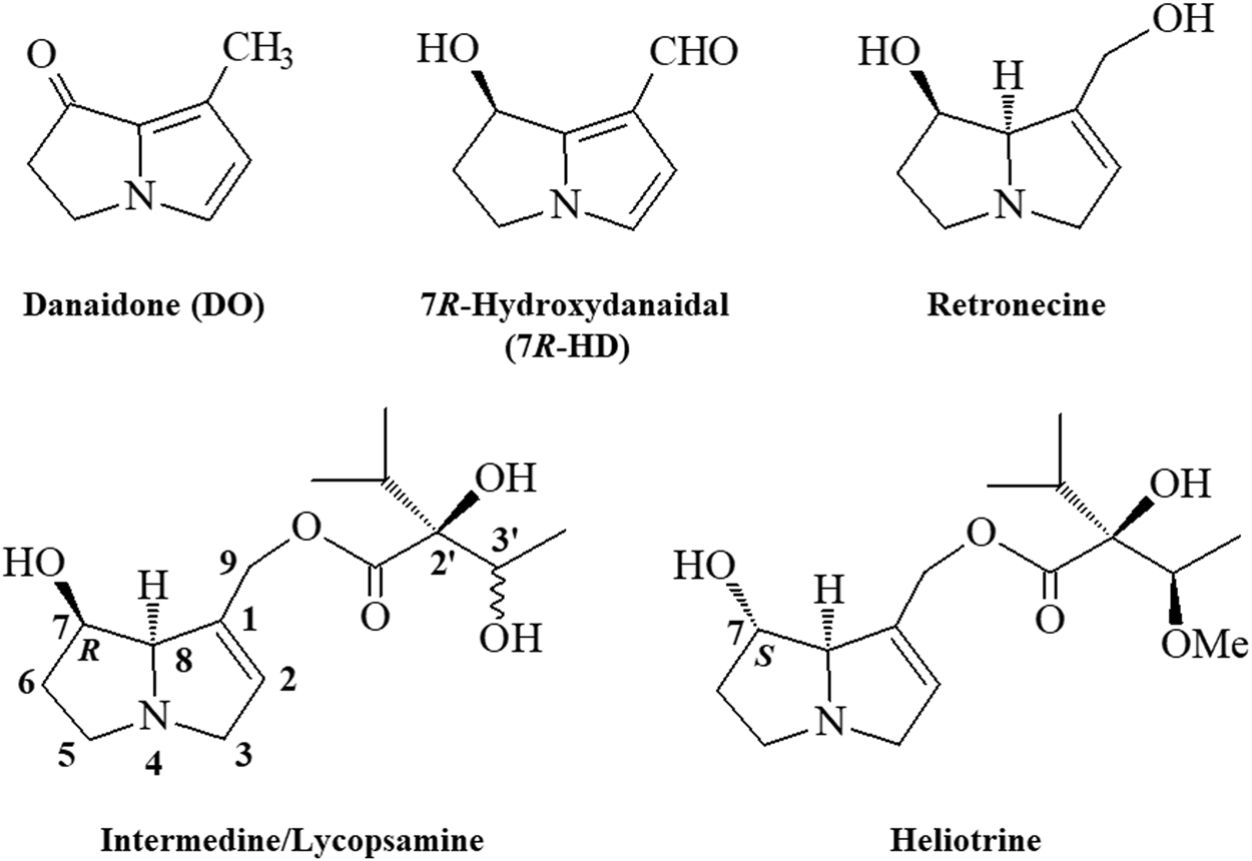
Chemical structures of the sex pheromone components of *Parantica sita* males and PAs that were orally administered to the males. A mixture of danaidone (DO, major) and 7*R*-hydroxydanaidal (7*R*-HD) constitutes the male sex pheromone of the butterfly. The stereochemistry at C3’ of intermedine and lycopsamine is *R* and *S*, respectively.

**Figure 2:**
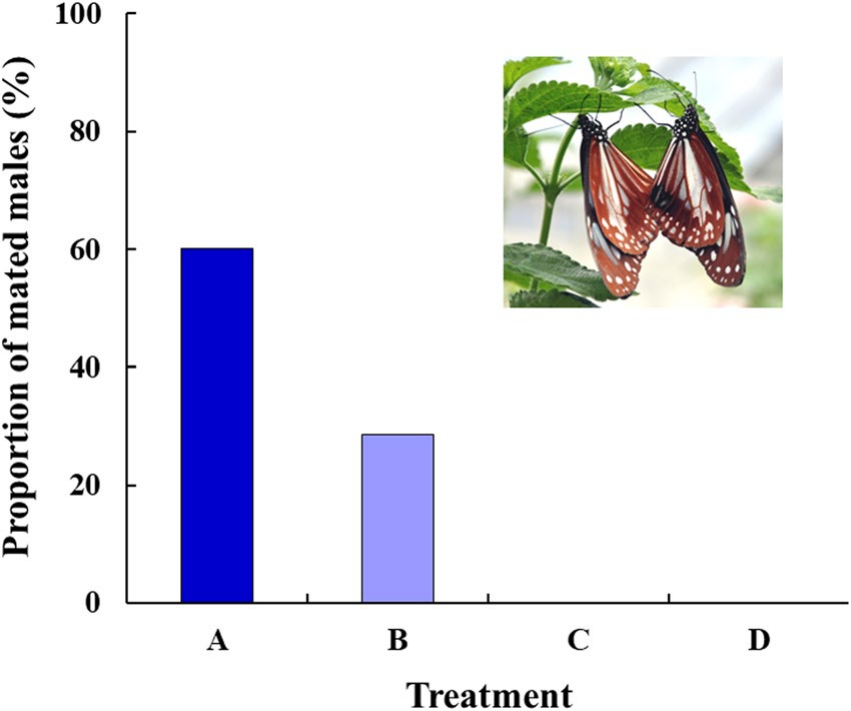
Mating success of *Parantica sita* males fed with different PA substances. The substances and their total quantities per male fed to the four cohorts were as follows: A, I/L (1.5 mg); B, I/L (0.5 mg); C, heliotrine (1.5 mg); and D, the hydrolysate mixture of I/L (1.5 mg). The numbers of males per cohort and females potentially engaged in the mating behaviour at any time point during the experiment, were 5 and 16, respectively. The proportion of mated males in A differed significantly from that in C and D (*p* < 0.05). The photograph shows a pair *in copula* (left, female; right, male).

The chemical structures of the two sex pheromone components, DO and 7*R*-HD, of *P. sita* males and PAs fed to the males are shown in Fig. 1. We first tested the effect of ingestion of some PA-related substances on male mating success (Fig. 2); PA substances administered to the males were a mixture composed of intermedine and lycopsamine (ca. 4:1, hereafter referred to as I/L), heliotrine, and a hydrolysate mixture of I/L. Of the four cohorts, only I/L-fed males (A and B) succeeded in mating, with a higher dose of I/L tending to facilitate mating; 60% of males (6 of 10) that were fed 1.5 mg of I/L (A) achieved copulation, while the copulation rate of those fed 0.5 mg of I/L (B) was only 28.6% (2 of 7). By contrast, none of the males that were fed heliotrine (1.5 mg, C) or a hydrolysate mixture of I/L (1.5 mg, D) were able to copulate. The copulation rate in A was significantly different from that in C or D (*p* = 0.044). Supplementary Table S1 shows the amount of DO retained in the hairpencils of individual males on the day following copulation or termination of the experiment. The pattern of DO possession by males was more or less similar among the cohorts; the presence of DO was confirmed in half or more of the males in all of the cohorts and the proportion of DO possession in mated males (50%; 4 of 8) was nearly the same as in unmated males (68.4%; 13 of 19), although *per capita* quantity of DO varied considerably.

### Effect of I/L ingestion on the mating success of males with the sex pheromone

**Figure 3:**
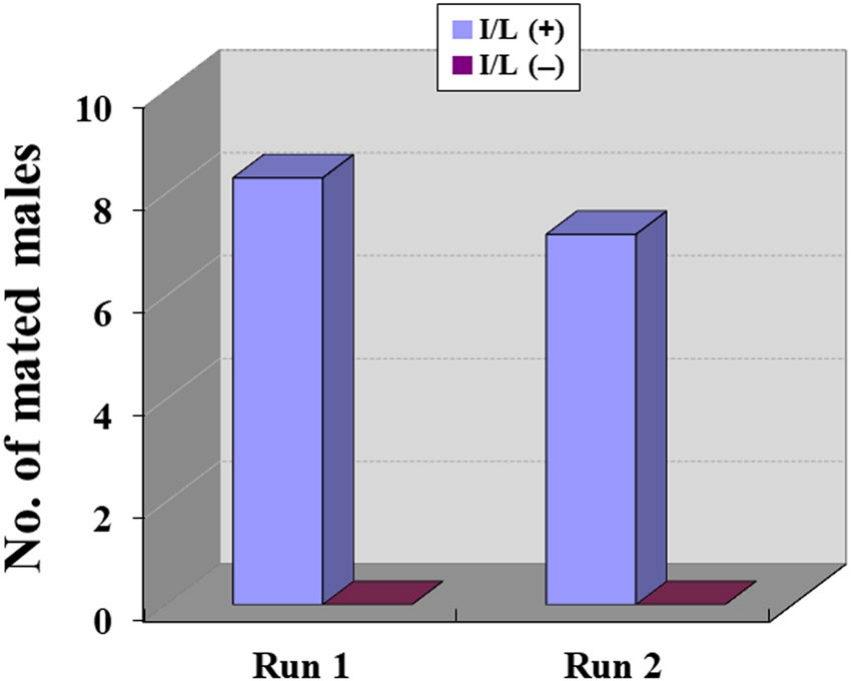
Mating competition between I/L-fed and I/L-unfed males of *Parantica sita*. All of the males had been maneuvered into possessing the sex pheromone before starting the experiment. Experiments were replicated twice. The numbers of I/L-fed males [I/L(+)], I/L-unfed males [I/L(−)], and females potentially engaged in the mating behaviour at any time point during the experiment for Runs 1 and 2, were 14/14/20 and 12/12/18, respectively. The difference in the copulation rate between I/L-fed and I/L-unfed males was significant (*p* < 0.0001).

**Figure 4:**
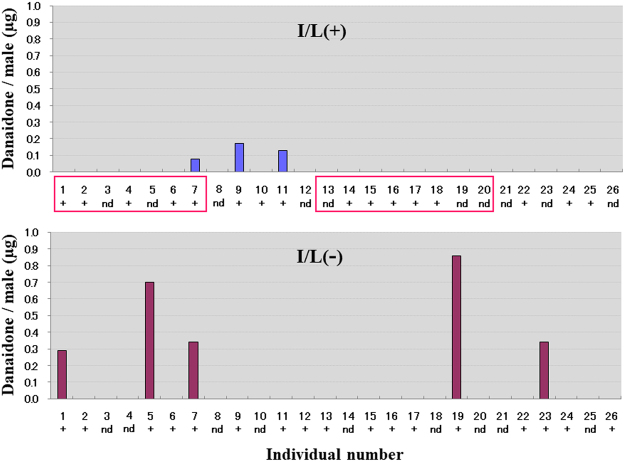
Individual variation of the amount of DO retained in the hairpencils of I/L-fed and I/L-unfed males of *Parantica sita* that participated in the mating competition. Data obtained from Runs 1 and 2 (Fig. 3) are collectively shown. DO determination was conducted on the day following copulation for mated males or on the day following termination of the experiment for unmated males. Stock males kept for replacement were also used for DO measurement, where appropriate. In cases where DO content was too low to quantify on GC, its presence (+) was confirmed by MS. nd, not detected. This, however, does not necessarily mean that the males in question were totally devoid of DO at the commencement of the experiment. The individual numbers in red squares denote males that achieved copulation. Prior to the experiment, both I/L-fed and I/L-unfed males had been equally fed retronecine (1.5 mg each), which can be a common precursor for both sex pheromone components (DO and 7*R*-HD).

The results shown in Fig. 3 clearly indicate that only males that were fed I/L succeeded in mating. The copulation rates of I/L-fed males in runs 1 and 2 were 44.4% (8 of 18) and 43.8% (7 of 16), respectively. The difference in the copulation rate between I/L-fed and I/L-unfed males was significant (*p* = 0.0000412). The patterns of DO possession in the hairpencils of both I/L-fed and I/L-unfed males, which were examined on the day following copulation or termination of the experiment, are shown in Fig. 4, with nearly the same proportions of males possessing DO in the two cohorts; in the I/L(+) cohort, the rate of males in which DO presence was confirmed was 61.5% (16 of 26), while that in the I/L(−) cohort was 65.4% (17 of 26). As in the preceding experiment (Supplementary Table S1), individual variation of the amount of DO was remarkable, particularly in the I/L(−) cohort. Interestingly enough, the amount of DO found in the hairpencils of the mated males was very small after copulation.

### Effect of I/L ingestion on the courtship activity of males without the sex pheromone

**Figure 5:**
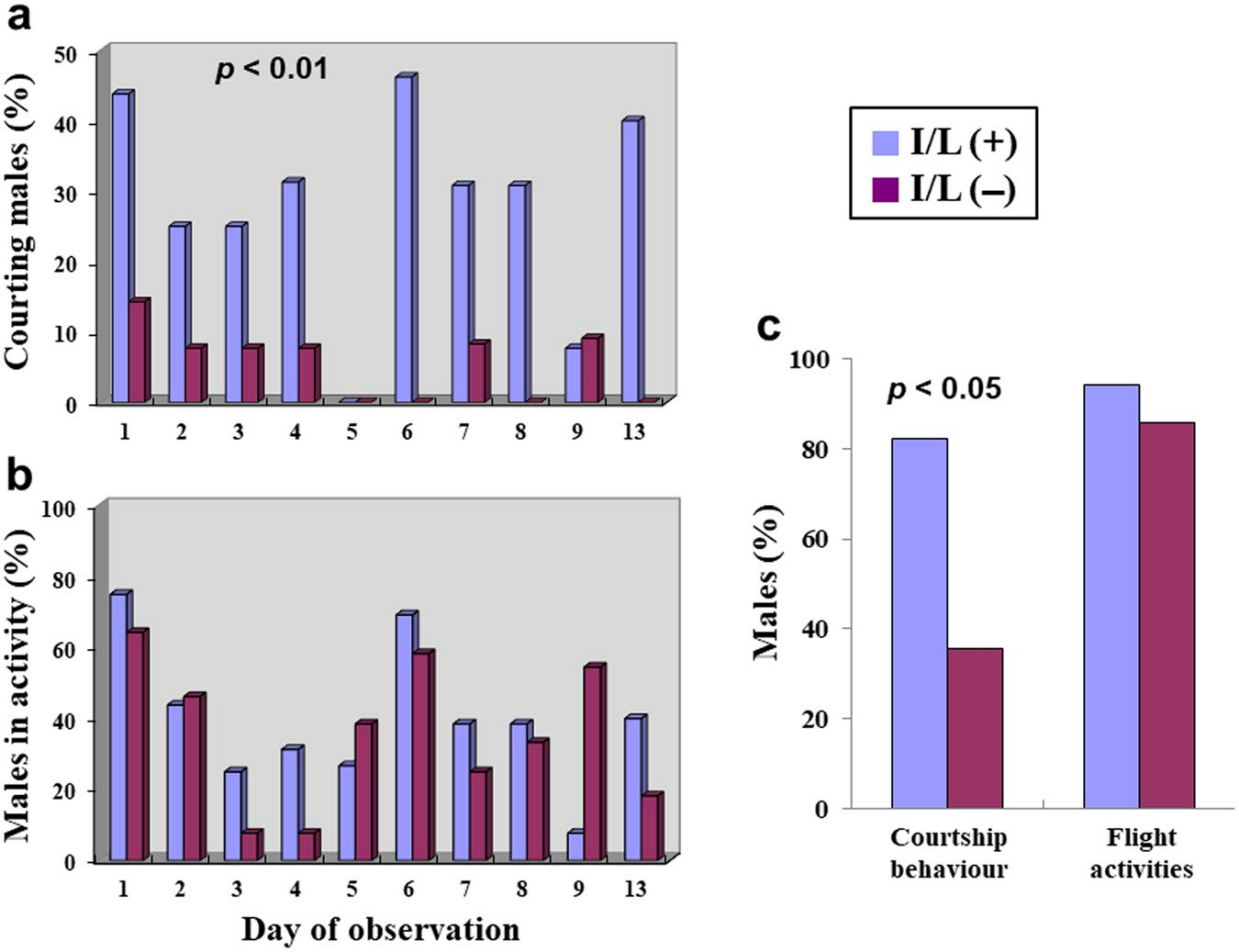
Behavioural patterns of I/L-fed and I/L-unfed males of *Parantica sita*. All of the males were devoid of the sex pheromone in the hairpencils owing to ablation of the sex brands prior to I/L administration. On days 10–12, observations were not made owing to rainy and stormy weather. (**a**) Percentage of males that displayed aerial pursuit of a female (courtship behaviour) at least once during each 1.5-hr observation period of the day. A significantly higher proportion of I/L-fed males [I/L(+)] courted females when compared with I/L-unfed males [I/L(−)] (*p* < 0.01). (**b**) Percentage of the sum total of males that engaged in flight activities including pursuit, foraging or patrolling during each 1.5-hr observation period of the day. Resting males were not included. (**c**) Percentage of males that were observed to exhibit courtship behaviour or flight activities including pursuit, foraging or patrolling at least once during the whole experimental period. Eventually, the numbers of I/L-fed and I/L-unfed males available for data acquisition were 17 and 14, respectively. The proportion of males that courted females was significantly higher in I/L-fed males than in I/L-unfed ones (*p* < 0.05).

Only two individuals of I/L-fed males achieved copulation, while none of the control (I/L-unfed) males did. Apparently, significantly larger number of I/L-fed males courted females when compared with control males (U = 41.5, z = 3.11, *p* = 0.0019) (Fig. 5a). At the same time, no significant difference in the sum total of males that performed flight activities (including pursuit of a female, foraging, or patrolling) was found between I/L-fed and control males (U = 101.5, z = 0.43, *p* = 0.6587) (Fig. 5b). Overall, the proportion of males that courted females at least once during the whole experimental period (Fig. 5c) was significantly higher in I/L-fed males (82.4%) than in control males (35.7%) (*p* = 0.01193), whereas as for the males that engaged in flight activities, the proportion of I/L-fed males (94.1%) did not differ significantly from that of control males (85.7%) (*p* = 0.5764).

### The level of biogenic amines in PA-fed and PA-unfed males

**Figure 6:**
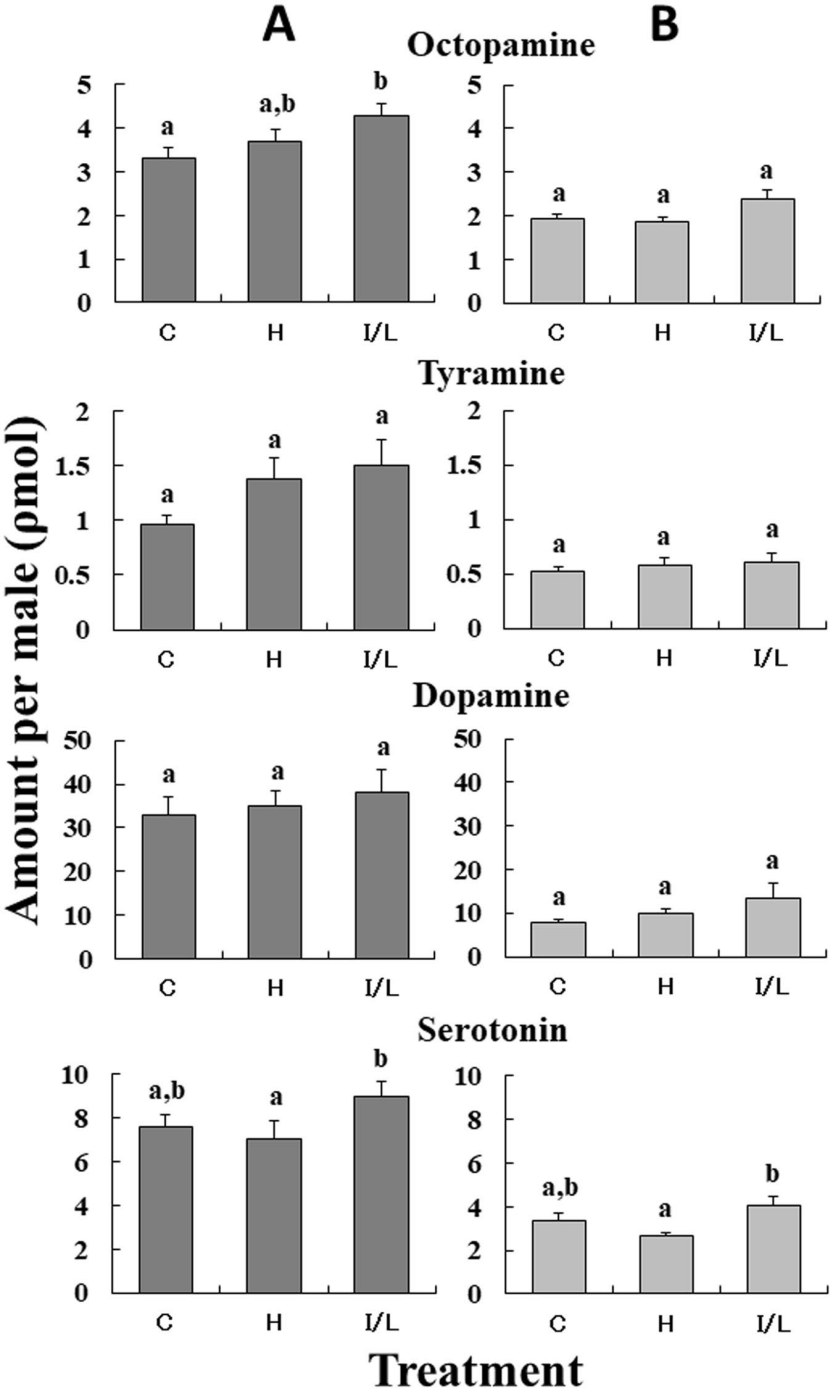
Amounts of biogenic amines (mean + SE) present in the brain (**A**) and thoracic ganglia (**B**) of *Parantica sita* males that were fed heliotrine (H; 1.5 mg each, *N* = 32) or I/L (1.5 mg each, *N* = 37 for brain and *N* = 35 for thoracic ganglia). C: control (PA-unfed) males (*N* = 35). Values unmarked by the same letters (in the same chart) are significantly different (*p* < 0.05 for octopamine and serotonin).

Four typical biogenic amines, viz. octopamine (OA), tyramine (TA), dopamine (DA), and serotonin (5-HT), present in the brain (Fig. 6A) and thoracic ganglia (Fig. 6B), were quantified. Of the four amines, DA was most abundant in both the brain and thoracic ganglia, with its content being approx. one order of magnitude higher than those of the other amines. It is noteworthy that males fed with I/L showed the highest average of the content of each amine in both the brain and thoracic ganglia; for example, the amine levels in the brain of I/L-fed males for OA, TA, DA, and 5-HT were 4.28 ± 0.26 (p mol, mean ± SE), 1.51 ± 0.23, 37.9 ± 5.40, and 8.98 ± 0.69, respectively, while those of the control for OA, TA, DA, and 5-HT were 3.32 ± 0.23, 0.96 ± 0.08, 33.1 ± 3.83, and 7.59 ± 0.59, respectively. In one-way ANOVA tests, significant differences in the amine quantities among the three cohorts were found from OA (*F* = 3.156*, p* = 0.0468) and 5-HT (*F* = 3.576*, p* = 0.0316) in the brain and also from 5-HT (*F* = 3.723*, p* = 0.0276) in the thoracic ganglia, while the levels of TA (*F* = 2.854*, p* = 0.0628) and DA (*F* = 0.158*, p* = 0.8543) in the brain, and those of OA (*F* = 2.133*, p* = 0.1239), TA (*F* = 0.199*, p* = 0.8196), and DA (*F* = 2.121*, p* = 0.1253) in the thoracic ganglia were not statistically significant. Subsequent post-hoc tests revealed that the difference in OA quantity in the brain between I/L-fed males and the control was significant (*p* = 0.04107). Furthermore, the levels of 5-HT in both the brain and thoracic ganglia of I/L-fed males were significantly higher than those of males fed with heliotrine (*p* = 0.02501 for brain and *p* = 0.02098 for thoracic ganglia).

## Discussion

Our previous studies^21,34^ have shown that three PAs, viz. I/L, heliotrine, and retronecine (a component of the hydrolysate mixture of I/L) (Fig. 1), can serve as biosynthetic precursors for *P. sita* males to produce DO. Of these, I/L and retronecine can also be precursors for 7*R*-HD. It is apparent that the chemical structure and dose of PAs profoundly affect the mating success of males (Fig. 2). Only I/L was effective in achieving copulation, despite the possession status of DO being similar among the four cohorts (Supplementary Table S1). Although it was impossible to determine the quantity of DO present in the hairpencils prior to the experiment (DO analysis inflicts lethal damage on the males), our previous data^21^ suggest that almost all of the males shown in Supplementary Table S1 possessed at least DO in varying amounts at the start of the experiment.

PAs are composed structurally of two subunits, viz. necic acid and necine base moieties. It should be noted that the necine base moiety of I/L is virtually composed of retronecine, which is a common ingredient of the two necine base-derived sex pheromone components (DO and 7*R*-HD) of *P. sita* males. Although we failed to determine the titer of 7*R*-HD because of its low content, these results strongly suggest that mere possession of the sex pheromone may not suffice for male mating success and that the mating success most likely depends on the chemical structure of PA(s) they ingest rather than on the quantity of pheromone they store.

We thus set out to clarify how I/L uptake influences the courtship behaviour and copulation of males in the presence or absence of the sex pheromone. The results of mating competition between I/L-fed and I/L-unfed males (Figs 3 and 4) definitely indicate that I/L-unfed males cannot accomplish copulation at all, even if they retain the sex pheromone. Given that both I/L-fed and I/L-unfed males had been equally fed 1.5 mg of retronecine prior to the onset of behavioural assay, the results reconfirm that retronecine as one of PAs is entirely ineffective in mating, and further indicate that additional ingestion of PA(s) with specific structures, such as I/L, is essential for males to attain copulation. Making a rough estimate, it appears that I/L-fed males tended to have slightly smaller amounts of DO than did I/L-unfed males (Fig. 4). This phenomenon may be, in part, a result of more frequent hairpencilling by I/L-fed males during their aerial interaction with females, because as both DO and 7*R*-HD are volatile, DO would be lost gradually each time males extrude their hairpencils.

The experiments with males lacking the sex pheromone (Fig. 5) evidently indicate again that I/L-fed males much more actively courted females than did control (I/L-unfed) males, thereby clearly indicating that male mating vigor has a close relationship simply with I/L uptake but not with the possession of the sex pheromone. Our observations of their courtship behaviour also revealed that most I/L-fed males were persistent suitors, chasing a female for more than 10 sec and occasionally exhibiting a circling flight around the female^21^, while those denied access to PAs tended not to pursue a female with persistence.

With regard to the biogenic amines of *P. sita* (Fig. 6), the level of brain OA in I/L-fed males was significantly higher than in control males, while the levels of 5-HT in both the brain and thoracic ganglia of I/L-fed males differed significantly from those of heliotrine(H)-fed males. In insects, OA has been shown to serve as a neurotransmitter, neuromodulator, and neurohormone in the nervous system^38,39^ and to prompt the whole organism to dynamic action^40–42^. In the peripheral nervous system, OA modulates the activity and energy metabolism of flight muscles, peripheral organs, heart, and almost all sense organs^41,43,44^. Regarding mating behaviours in males, the brain OA can produce the basic activity of the pattern generator for copulation actions in the cricket^45^ and enhance the behavioural response of oriented flight to sex pheromone in the moth *Agrotis ipsilon*^46^. In male honey bees, OA has been shown to promote their mating flight activities^47^. OA is also released from DUM neurons in the terminal abdominal ganglion to the male reproductive organ, including accessory glands, epididymis and ejaculatory duct^48^, suggesting the involvement of OA with formation of spermatophore. At the same time, in moths and the honey bee, 5-HT is thought to operate in primary olfactory centers of the brain, and function as a regulator of neuronal development and a mediator of cellular and behavioural plasticity, increasing the excitability of central olfactory neurons^49–51^. It seems, therefore, likely that specific PA(s), i.e. I/L for instance, directly or indirectly stimulated the nervous system to elevate the levels of certain biogenic amines in the brain and thoracic ganglia, resulting in enhancing the mating motivation and ability of I/L-fed males, which in turn facilitated their mating as a consequence.

All these results taken together demonstrate that although the sex pheromone can be regarded as a requisite, enhancement of mating motivation through acquisition of specific PA(s) is also imperative for *P. sita* males to achieve smooth copulation. In other words, PA(s) with particular structural features, e.g. I/L, are considered to play a dual role of precursors for the sex pheromone and activators for the courtship behaviour. In an arctiid moth, *Utetheisa ornatrix*, the titer of its male sex pheromone (7*R*-HD) that he produces from PA(s) (for example, monocrotaline) acquired as a larva from its food plants, is thought to have the potential to advertise male’s worth, viz. his intrinsic PA load (systemic PA content) that can directly communicate his state of chemical defendedness and his PA-donating capacity to females^52^. In another arctiid moth, *Estigmene acrea*, monocrotaline *N*-oxide fed to larvae has been shown to stimulate males to display their androconial organs (coremata)^53^.

What structural features do specific PAs have, then? As far as *P. sita* is concerned, at least lycopsamine-type PAs (lycopsamine, intermedine, and indicine) fall into this category. One of the most important facets worthy of attention is that not all PAs equally stimulate feeding; some PAs serve as phagostimulants while others deter feeding. More importantly, this tendency is closely related to their availability in pheromone production^34^. To our knowledge, male *P. sita* display the strongest preference for I/L, which is also the best precursor for pheromone biosynthesis^21,34^. In fact, PAs found from plants that male *P. sita* preferentially visits, are lycopsamine, intermedine, and indicine^21^. Consequently, it follows that the structure of necic acid moiety of PAs they ingest critically affects their reproductive strategies.

Pharmacophagy has hitherto been discussed mainly from the viewpoint of chemical defense against natural enemies or pathogenic microbes and of biosynthetic precursors for pheromones. Our results, however, present new evidence for the evolutionary provenance of pharmacophagy that acquisition of specific PA(s) are inevitably involved in the mating success of male *P. sita*, presumably via enhanced mating activity stimulated by the alkaloid. Although close associations between danaines and PAs seem to stem from the exploitation of PA-containing plants as hosts (putative ancestral host plants^54,55^), the present-day pharmacophagous utilization of the alkaloid by adult danaine butterflies that has persisted ever since host shifts from PA plants to non-PA ones^54^, is deemed to reflect an exceptional role for specific PA(s) in their reproductive strategy.

## Methods

The experimental design for behavioural bioassays was strictly based on the findings presented in our previous paper^21^ that reported 1. DO and 7*R*-HD are first produced in the sex brand and at least DO is subsequently physically transferred to the hairpencil through the perfuming behaviour (contact behaviour between the two androconial organs), 2. if the sex brands are wholly ablated, the two sex pheromone components are no longer accumulated in the hairpencils, and 3. however, ablation of the sex brands does not affect subsequent mating success of males, as long as the hairpencils, after the perfuming behaviour, can carry the sex pheromone in an amount sufficient for courtship.

### Insect

Adults of *P. sita* subjected to various experiments were offspring of wild females captured in Hiroshima, Kochi, and Kagoshima prefectures. Larvae were reared on potted plants of their hosts, *Marsdenia tomentosa*, *Tylophora tanakae* or *Cynanchum japonicum* (Apocynaceae, Asclepiadoideae), under standard laboratory conditions (16L-8D, 24–25 °C). Adults, which were sexed immediately after emergence, were kept separately in transparent plastic chambers (25 × 35 cm; height, 21 cm) at 25 °C under a 16L-8D regime (ca. 100 lux), and fed 15% aq. sucrose solution once daily until the commencement of experimental programs.

### Preparation of hairpencil extracts and determination of DO

In experiments during which copulation took place, pairs *in copula* were carefully transferred to the laboratory. On the day following copulation, the amount of DO in the hairpencils retained by the mated males was examined. In addition, the amount of DO in the hairpencils was also determined for unmated males, where necessary, on the day following termination of the experiment.

The hairpencils were artificially protruded with forceps, excised, and extracted individually with 100 μl of purified dichloromethane. The extracts were stored at −20 °C until use. Chemical analyses of the extracts were performed by gas chromatography (GC) and gas chromatography-mass spectrometry (GC-MS). GC analyses were carried out on a Shimadzu GC-14A gas chromatograph equipped with a flame-ionization detector, using an EQUITY-1 fused-silica capillary column (Supelco, 0.25 mm I.D. × 15 m, 0.25 μm film thickness). Samples were injected splitless at 250 °C using N_2_ as the carrier gas (flow; 1 ml/min) and the oven temperature was programmed from 50 °C (held initially for 5 min) to 300 °C (held for 10 min) at 10 °C/min. EI-MS spectra were recorded at 70 eV on a Shimadzu GCMS-QP5000 mass spectrometer coupled with a Shimadzu GC-17A gas chromatograph, using a DB-1 fused-silica capillary column (J&W, 0.25 mm I.D. × 15 m, 0.25 μm film thickness) and He as the carrier gas (flow; 1 ml/min). The GC system was operated with the same temperature program as that employed in GC analyses. Identification of DO was based on a comparison of its mass spectral and GC retention data with those of authentic samples. Quantification of DO was performed by GC using benzyl alcohol as the internal standard. The absolute amount of DO was determined on the basis of a calibration curve made with authentic DO and benzyl alcohol. However, in the case where DO content was too low (below *ca*. 10 ng per male) to quantify on GC, its presence was examined by checking the molecular ion (*m/z* 135) of DO in MS.

### Chemicals

Monocrotaline (Aldrich, purity: 99%) and heliotrine (Latoxan, 98%) were commercially purchased. A PA mixture I/L (a ca. 4:1 mixture of intermedine and lycopsamine, 95% as a whole) was obtained from the roots of *E. glehnii* collected in Hiroshima Pref. according to the method given in our previous paper^56^. Retronecine (98%) was prepared from monocrotaline by the reported method^57^. A hydrolysate mixture of I/L, which comprised retronecine, trachelanthic acid, and viridifloric acid (molar ratio, 1.0:0.8:0.2), was prepared by hydrolyzing I/L with 3% aq. ammonia at room temperature. DO (99%) was prepared by the method of Rajaraman & Jimenez^58^, and purified by repeated sublimation at 40 °C under reduced pressure.

### Oral administration of PAs

When necessary, males were fed with a given quantity of any one of the PA compounds or PA-related substances dissolved in distilled water, by applying droplets of the solution to the coiled proboscis with a microsyringe.

### Behavioural bioassay to test the influence of structural types of PAs ingested on male mating success

Seven days after emergence, males were randomly divided into four cohorts (A-D), each of which consisted of five individuals. Thereafter, each cohort was fed daily with a given dose of a predetermined PA substance for 3 days. The PA substances and their total quantities per male fed to the cohorts A, B, C, and D were I/L (1.5 mg), I/L (0.5 mg), heliotrine (1.5 mg), and the hydrolysate mixture of I/L (1.5 mg), respectively. On post-emergence day 10, males of all the cohorts (20 males in total) were released, together with 16 females, into an outdoor cage (7 m × 10 m, 3.5 m high) equipped with PA-lacking flowers as a nectar source. A mating competition was staged among the four cohorts, maintaining the number of males of each cohort at five, while keeping the ratio of total males to females at ca. 4 to 3 throughout the experiment. Since copulation takes place mostly in the late afternoon and lasts overnight^21^, pairs *in copula* were collected between 18:00 and 18:30 every day. Individuals that had copulated, died, or lost sufficient flight ability during the experiment were replaced with new ones. The contest was carried on for 22 consecutive days.

### Effect of I/L ingestion on the mating success of males with the sex pheromone

After 7 days of emergence, males were fed daily with 0.5 mg of retronecine for 3 days (1.5 mg/male). On the following day, males were released into the same outdoor cage as described above, where they freely spent several days and were allowed to perform the perfuming behaviour. On day 17 of emergence (at this time point, all of the males are supposed to have the sex pheromone in the hairpencils^21^), both sex brands of all the males were ablated (thereafter, males can no longer produce additional amounts of the sex pheromone) and the males were evenly divided at random into two cohorts, the treated and the control. The treated males (*N* = 14) were fed daily with 0.5 mg of I/L for 3 days (1.5 mg/male), while control males (*N* = 14) were fed with 15% aq. sucrose only. Subsequently, both treated and control males were released again into the cage together with 20 females, and permitted to compete for mates for 18 consecutive days. Pairs *in copula* were collected between 18:00 and 18:30 every day. Individuals that had copulated, died, or lost sufficient flight ability were replaced with new ones, where necessary, so that the ratio of males between the treated and the control could be maintained at 1 to 1, while the ratio of males to females was maintained at ca. 4 to 3 throughout the experiment. The same experiment as above was replicated once more using 12 treated males, 12 control males, and 18 females.

### Effect of I/L ingestion on the courtship activity of males without the sex pheromone

As our previous study^21^ has revealed that males with no sex pheromone can seldom attain copulation, we focused our attention on the courtship activity exhibited by males in this experiment. Within 48 hr of emergence, both sex brands of males (*N* = 40) were ablated (by this treatment, the sex pheromone never occurs in the hairpencils^21^). After 7 days of emergence, half of the males (treated) were fed daily with 0.5 mg of I/L for 2 days (1.0 mg/male) and the rest (control, *N* = 20) were fed daily with 15% aq. sucrose only. Thereafter, both treated and control males were released into the same cage together with 30 females. Male courtship behaviour was observed from 17:00 until 18:30 every day for 10 days, during which the ratio of males to females was in the range between 4:3 and 3:2. Because *P. sita* males exhibit a characteristic pursuit of females in the incipient phase of the courtship^21^, we recorded male aerial pursuit of a female that lasted for more than 10 sec as reliable evidence for male mating vigor. We also observed ordinary daily activities of males, such as foraging and patrolling.

### Sample preparation for biogenic amine determination in PA-fed and PA-unfed males

After 7 days of emergence, males were fed daily with 0.5 mg of I/L (*N* = 37) or heliotrine (*N* = 32) for 3 days (1.5 mg/male). Control males (*N* = 35) were fed daily with 15% aq. sucrose only. Thereafter, all of the males were fed 15% aq. sucrose solution once a day, and kept in plastic chambers (25 × 35 cm; height, 21 cm) at 25 °C, which were externally illuminated daily with an incandescent lamp (ca. 3500 lux) for a few (typically 3) hr in the afternoon to stimulate their flight activity. On post-emergence day 20 ± 1, the brain and thoracic ganglia (pro-, meso-, and meta-thoracic ganglia were treated together) of each male were excised, and immediately stored separately and individually in liquid N_2_ until use.

### Measurements of biogenic amines in the brains and thoracic ganglia

Frozen brains and thoracic ganglia were dissected in ice-cold phosphate buffer (pH 7.0) on a Peltier cooling unit (Kenis Ltd., Osaka, Japan) at approximately 4 °C under a microscope. Dissected brains and thoracic ganglia were homogenized with a microglass homogenizer in 50 μl of ice-cold 0.1 M perchloric acid containing 100 ng/ml 3,4-dihydroxyphenylacetic acid (DHBA) for 2 min. Each sample was then transferred into a 1.5-ml microcentrifuge tube and centrifuged at 15,000 rpm for 30 min at 4 °C. Supernatants were transferred to microvials for analysis by high-performance liquid chromatography with electrochemical detection (HPLC-ECD).

For the analysis of biogenic amines, a HPLC-ECD system that was developed by Mezawa *et al*.^47^ was applied. The HPLC system comprised a solvent delivery pump (EP-300, EICOM, Kyoto, Japan), a refrigerated automatic injector (231–401, Gilson, Middleton, WI, USA) and a C18 reversed-phase column (250 mm × 4.6 mm id., 5 μm average particle size, UG 120, Shiseido, Japan) maintained at 35 °C. An electrochemical detector set at 0.8–0.9 V was used under 35 °C. Signals from the electrochemical detector were recorded and integrated by using data analysis software (PowerChrom, ADInstrument, Castle Hill, NSW, Australia). The mobile phase contained 0.18 M monochloroacetic acid and 40 μM 2Na-EDTA, which was adjusted to pH 3.6 with NaOH. Into this solution, 1.62 mM of sodium-1-octanesulfonate and 6.5% CH_3_CN were added. The flow rate was kept constant at 0.7 ml/min. External standards were run before, midway through and after the sample runs for the identification and quantification of OA, TA, DA and 5-HT. In the HPLC-ECD system, each biogenic amine peak was identified by comparing both the retention time and hydrodynamic voltammograms with those of the standards. Measurements based on peak area of the chromatograms were obtained by calculating the ratio of the peak area of a substance to the peak area of the internal standard.

### Statistics

Statistical analyses were performed with an EZR software (Saitama Medical Center, Jichi Medical University, ver. 3.3.2). All *p* values were two-sided. The qualitative and quantitative effects of PA substances on male mating success were assessed by a Fisher’s Exact test (FET) with adjustment by the Bonferroni method. FET was used also for the comparison of the copulation rates of males that possessed the sex pheromone and for the appraisal of the effects of I/L uptake on the courtship behaviour of males without the sex pheromone. The daily records of the courtship behaviour and flight activities of males that were devoid of the sex pheromone were compared between I/L-fed and I/L-unfed males and analyzed by a Mann-Whitney *U*-test. The significance of difference in the levels of biogenic amines present in the brain and thoracic ganglia was tested by one-way ANOVA, in which all the raw data had undergone logarithmic transformation (by this treatment, the distribution of the original data approached the normal distribution), followed by the post-hoc test (Tukey-Kramer test) for multiple comparisons.

### Data Availability

The datasets generated during and/or analyzed during the current study are available from the corresponding author on reasonable request.

## Electronic supplementary material

SUPPLEMENTARY INFORMATION
