# Supplementary material for: Uptake of plant-derived specific alkaloids allows males of a butterfly to copulate
Source: Sci Rep. 2018 Apr 3;8:5516. doi: 10.1038/s41598-018-23917-y (PMC5882650; doi:10.1038/s41598-018-23917-y)
Supplement: Supplementary file 1 — SUPPLEMENTARY INFORMATION [file 41598_2018_23917_MOESM1_ESM.docx]

**SUPPLEMENTARY INFORMATION**

**Uptake of plant-derived specific alkaloids allows males of a butterfly to copulate**

Keiichi Honda^1,3*^**,** Junya Matsumoto^1^, Ken Sasaki^2^**,** Yoshiaki Tsuruta^1^, and Yasuyuki Honda^1^

^1^Department of Biofunctional Science and Technology, Graduate School of Biosphere Science, Hiroshima University, Higashihiroshima 739-8528, Japan

^2^Graduate School of Agriculture, Tamagawa University, Machida 194-8610, Japan

^3^Present address: Saijo Ecology Institute, 1387-38 Iida, Hachihonmatsu

Higashihiroshima 739-0141, Japan

^*^Corresponding author: [cehonda@kamon.ne.jp](mailto:cehonda@kamon.ne.jp)

Figure S1. Males of *Parantica sita* (Nymphalidae, Danainae) congregating on and feeding at decayed parts of *Messerschmidia sibirica* (Boraginaceae) that grows on the sandy coast. This plant is relatively rich in pyrrolizidine alkaloids composed mainly of lycopsamine and intermedine, which the males prefer to ingest and utilize to produce their sex pheromone. Photographed by Norio Daikai on May 31, 2014 on Himeshima Island, Oita prefecture, Japan.

DO determination was conducted on the day following copulation for mated males or on the day following termination of the experiment for unmated males.

The individual number in red denotes a male that achieved copulation.

In the case where DO content was too low to quantify on GC, its presence (+) was confirmed by MS. nd, not detected. This, however, does not necessarily mean that the males in question were totally devoid of DO at the commencement of the experiment.
